# Predicting sustainable fashion consumption intentions and practices

**DOI:** 10.1038/s41598-024-52215-z

**Published:** 2024-01-19

**Authors:** Yingxiu Hong, Abdullah Al Mamun, Qing Yang, Mohammad Masukujjaman

**Affiliations:** 1 Business School, Nanfang College Guangzhou, Guangzhou, 510970 Guangdong China; 2 UCSI Graduate Business School, UCSI University, Kuala Lumpur, Malaysia; 3 UKM-Graduate School of Business, Universiti Kebangsaan Malaysia, 43600 Bangi, Selangor Darul Ehsan Malaysia; 4 Faculty of Business Management and Professional Studies, Management and Science University, 40100 Shah Alam, Selangor Malaysia

**Keywords:** Environmental social sciences, Human behaviour

## Abstract

The fashion industry has a significant impact on the environment, and sustainable fashion consumption (SFC) has become a pressing concern. This study aimed to investigate the factors influencing sustainable fashion consumption behavior (SCB) among Chinese adults, specifically the role of values, attitudes, and norms in shaping such behavior, using the value-belief-norm framework. The study used an online cross-sectional survey design to collect data from 350 participants recruited through a convenience sampling method using social media platforms and email invitations, and the obtained data were analyzed using partial least squares structural equation modelling. The results of the study showed that biospheric (BV), altruistic (AV), and egoistic (EV) values significantly influenced the New ecological paradigm (EP), which, in turn, positively affected awareness of consequences (AC). Personal norms (PN) were positively influenced by EP, AC, and ascription of responsibility (AR). Social norms (SN) and trust in recycling (TR) were also found to positively influence sustainable fashion consumption intentions (SCI). Finally, the study found that SCI and TR were significant predictors of SCB, whereas the moderating effect of TR not statistically significant. The study’s originality lies in its comprehensive investigation of the interplay between various factors (particularly using norms in two facets; PN and SN) in shaping SCB, using a structural equation modeling approach, and exploring the moderating effect of TR. The findings of this study suggest that interventions aimed at promoting SFC should focus on fostering values and beliefs that prioritize the environment, encouraging individuals to take responsibility for their actions, creating an environment in which SFC is normalized, and increasing TR.

## Introduction

The issue of global environmental pollution is exacerbated by unsustainable consumer practices, such as the excessive or one-time acquisition of clothing items^1^. China, the world's largest textile manufacturing nation, grapples with textile and garment production waste exceeding 100 million tons^2^ and an annual disposal of roughly 26 million tons of used clothing, projected to rise to around 50 million tons by 2030, with a recycling rate of less than 1%^3^. In response to these challenges, China, along with other countries, has committed to achieving "net-zero carbon emissions" by 2050^4^, which necessitates collaboration between the fashion industry and the general public. One of the key strategies in this context is the adoption of second-hand consumption^5^. Reintroducing pre-owned items extends product lifecycles, curbing the need for new items, conserving energy and resources, and significantly reducing the environmental impact tied to consumer behavior, emphasizing the crucial role of sustainable consumption^6^.

Despite certain advancements in the industry, it is noteworthy that China's volume of second-hand goods transactions amounted to just over half of that seen in the United States^1,7^. Similarly, the second-hand clothing-sharing market in China is still in an exploratory stage compared to the European market^1^. Given China's influence on traditional social hierarchy thinking, Chinese individuals tend to prioritize identity and status, which may lead to more rigid perspectives on second-hand items^8^. Chinese people often tend to be conservative, placing a premium on privacy, and may display some reluctance towards items from unfamiliar sources^1^. Nevertheless, with the global consensus on sustainable development, the sharing economy has gained increasing prominence among Chinese youth^9^. Simultaneously, government initiatives and state influence have promoted sustainable consumption across different segments of society^10^, potentially leading to a shift in the attitudes of consumers with rigid views on second-hand product sharing and trading. With its vast population and status as the world's largest clothing consumer, it underscores the untapped opportunities within China's second-hand clothing market and its potential for sustained expansion^11^. Therefore, platforms must evaluate present consumer considerations and identify the factors influencing consumption, enabling them to align with the right developmental trajectory.

Previous research has examined how mainstream consumers feel about and choose sustainable fashions. Environmental concerns and social norms (SN) are the main reasons why people buy sustainable fashion^12–14^. Conversely, recent research indicates an increased environmental consciousness among individuals, leading to a greater inclination to purchase eco-friendly products, including fast fashion items^15^, second-hand apparel^5^, reduced clothing consumption, and clothing recycling practices^16^. Other studies^17,18^ have found that some eco-conscious consumers prefer to buy sustainable fashion by purchasing eco-friendly brands or buying used clothing, and recycling, reusing, renting, or swapping clothing. Despite a link between people’s causes and ways of throwing away clothes, practitioners and policymakers know that expectations and reality are not the same regarding the use and disposal of fashion products^19^. Hur^20^ states that the majority of individuals are unaware of what happens to donated used apparel when it has reached the end of its useful life. Therefore, little attention has been paid to encouraging sustainable fashion consumption (SFC) through policy interventions or learning about how consumers reuse second-hand clothing. Thus, it is becoming increasingly important to understand what makes people want to buy sustainable fashion, and how they feel about reusing second-hand clothes.

Various theoretical frameworks have been used to determine sustainable behavior. Researchers have employed the Theory of Planned Behavior (TPB) and the Norm Activation Model (NAM) theories in various research contexts, as demonstrated in previous studies^21,22^. In recent years, an increasing number of researchers have utilized value-belief-norm (VBN) theory to predict environment-friendly behavior, and have validated its efficacy in different settings, such as recycling^23^, energy conservation^24^, and public support for green policies^25^. Other scholars have proposed extended versions of these theoretical frameworks by integrating key constructs from the TPB, VBN, and NAM into better models^22,26^. Yeap et al.^27^ studied the second-hand cloth purchase intention in Malaysia in the perspective of the customer-to-customer (C2C) online platform based on the Integrative Model of Behavioural Prediction. Lang and Armstrong^28^ focused on examining the adoption of cloth renting and swapping among female consumers, thereby allowing space for a broader understanding applicable to both male and female consumers overall. Recently, Zhang et al.^29^ utilized TAM and TPB as an integrated model to evaluate cloth disposal behavior in China. However, although Zahid et al.^30^ investigated second-hand cloth purchasing behavior in the Chinese context, they failed to establish any theoretical basis to guide readers. However, the use of VBN in second-hand clothing is limited. Gomes et al.^31^ recently used VBN theory in a comparative study between Brazil and the Netherlands, yet stressed its value and ignored the original model to a great extent. The VBN theory provides a distinct benefit when utilizing SFC by examining green behavior from the perspective of various essential components that are strongly linked to environmentalism, including values and ecological worldviews. The VBN theory addresses individuals' pro-social incentives by integrating rational-choice models that contain self-interest motivations related to environmentally friendly behavioral intents in various contexts^32^. Thus, the application of VBN with a couple of new variables may extend the understanding of the reuse behavior of second-hand clothing.

Although the TPB, VBN, and NAM theories have been used to measure environmental behavior in several studies, most have focused on single behaviors, such as recycling, household energy use, and green consumption, rather than multidimensional measures of environmental behavior. To the best of our knowledge, few studies have combined intentions and behaviors^33^. Davies and Gutsche^34^ suggested that little research has been conducted on how people actually buy things, which makes people wonder how much is known about green consumption practices. Therefore, there is a pressing need for a deeper understanding of the reasons and processes behind distinct consumer behaviors. Consumers often experience psychological deflation that leads them to shop recreationally to relieve boredom or stress, creating a psychological imbalance between their sustainability worries and buying sustainable fashion^35^. Sustainable fashion consumption behavior (SCB) is further hindered by the need to express a certain social identity^36^ and the absence of convenient and sustainable clothing options. Fast fashion is more readily available to customers than environmentally conscious apparel options that require more effort and time to track. Trust is also a significant factor in the transition from intention to behavior^37^, as hindering factors can prevent consumers from acting according to their initial intentions^38^. To narrow the gap between intention and behavior regarding second-hand clothing sharing, researchers should include trust issues as a moderating factor.

While many studies have been conducted on SFC in industrialized countries such as the US, Europe, and Asia, there is a noticeable lack of research on SFC in Asian countries^13,14,39^. In their comparative study, Su et al.^14^ conducted research in both the USA and China, utilizing the VBN framework and integrating constructs like apparel sustainability knowledge, consumer value, attitude, and willingness to purchase. Vehmas et al.^39^ interviewed Finnish consumers about their perceptions and attitudes towards circular clothing and the communication and marketing channels of second-hand clothing without using any behavioral frameworks. Similarly, Baier et al.^13^ sought answers about the drivers of pre- and post-purchase behavior in the German apparel and sports industry, using the segmented Kano method while excluding behavioral models. While studies such as Wang et al.^40^ and Zhang et al.^29^ addressed the issues of SFC from Chinese perspectives, the former analyzed the motivations and barriers to consumers' purchase of second-hand clothes, along with their perceived problems with this industry, without concentrating on adoption issues. In contrast, the latter study focused on another aspect of SFC, specifically cloth disposal behavior, with no indication regarding the adoption of second-hand cloth from customer perspectives. This is a significant gap, as SFC has become increasingly popular among consumers in these markets^41^ especially the second-hand clothes because of their preference for fashion options with lower environmental and social impacts. However, sustainable consumerism in developing countries may be affected by a variety of cultural and economic factors, including, but not limited to, varied ethical ideals in relation to environmentally friendly fashion and lower income levels^14^. Studies have shown that sustainability knowledge and consumer preferences differ across countries, highlighting the importance of understanding cultural and economic differences^42^. Therefore, it is crucial to investigate sustainable fashion consumption exclusively on the Chinese setting to gain insight into consumers’ perspectives on the reuse of second-hand clothing.

To fill these knowledge gaps, current study created a theoretical model that analyzes the factors influencing shoppers’ decisions to buy second-hand clothing in China. The VBN variables were used in these models, and SN and trust were included. The primary goals of this research are to test whether the proposed framework, which incorporates social norms and trust, has higher predictive power than the original VBN models; find the most influential constructs for discussing intention and behavior; and assess whether VBN factors greatly impact people’s ecological behavior in the setting of second-hand clothes. This research contributes to the expanding body of literature on SFC by building upon prior studies that examine individual environmental behavior. It incorporates the VBN framework and proposes a comprehensive model to offer a more comprehensive understanding of the disparity between intention and conduct in SFC. This study effectively demonstrates the predictive effects of bi-dimensional norms, namely PN and SN, in shaping SFC, through their incorporation within the VBN frameworks. As a result, this study substantially contributes to the current understanding of the impact of social norms on promoting environmentally friendly behavior. From a managerial standpoint, this study offers valuable guidance for managers, emphasizing the need to integrate environmental values, awareness, responsibility, and trust in recycling into comprehensive strategies. Furthermore, the study underscores the managerial significance of actively shaping social norms supportive of SFC through sustainable fashion events, collaborations, and blogs, providing actionable guidance for managers seeking to influence consumer behavior and foster a broader culture of sustainability within the fashion industry. However, this paper is structured as follows: within its second section, it presents a literature review, outlines the proposed model, and presents the research hypotheses. Methods such as sampling, data gathering, measurement, and data analysis are outlined in “Materials and methods”. The results are presented in “Findings”, and the results, their possible effects, the study’s limitations, suggestions for further research, and closing remarks are discussed in “Discussion”.

## Theoretical background and hypotheses development

According to Pencarelli et al.^43^, sustainable products offer environmental, societal, and economic advantages while safeguarding public health, welfare, and the environment throughout their entire commercial cycle, from raw material extraction to ultimate disposal, with a focus on meeting the needs of future generations. Similarly, Mohr et al.^44^ define responsible consumption as a purchasing and consumption behavior pattern that aims to maximize long-term benefits while minimizing harmful impacts on both consumers and societies. In this study, the term SFC indicates the reuse of clothes, such as buying or selling used clothes at a minimal price^18^, swapping used clothes^28^, or donating used clothes to others. Thus, according to Bianchi and Gonzalez^45^, consumers who cannot afford high-priced fashion may choose to consume sustainably by purchasing second-hand apparel from thrift stores or swapping clothes with their family or friends. They may sell clothes at minimum prices to stores or donate clothes to those in need from a philanthropic viewpoint.

### Theoretical foundation

VBN theory denotes that “individuals who accept a movement’s basic values, believe that valued objects are threatened, and believe that their actions can help restore those values, experience an obligation (personal norm) for pro-movement action”^46^. According to Schwartz’s^47^ theory, actions relevant to norms encompass three concepts: the acceptance of an individual’s particular values, the belief that something important to those values is under threat, and the belief that a person’s behavior can help alleviate the threat and restore value, which are known as personal norms. Schwartz^48^ classifies values into three types: biospheric, altruistic, and egoistic. Beliefs consist of new ecological paradigm (EP), awareness of consequences (AC), and ascription of responsibility (AR), which, in combination, affect behavioral intention.

**Figure 1:**
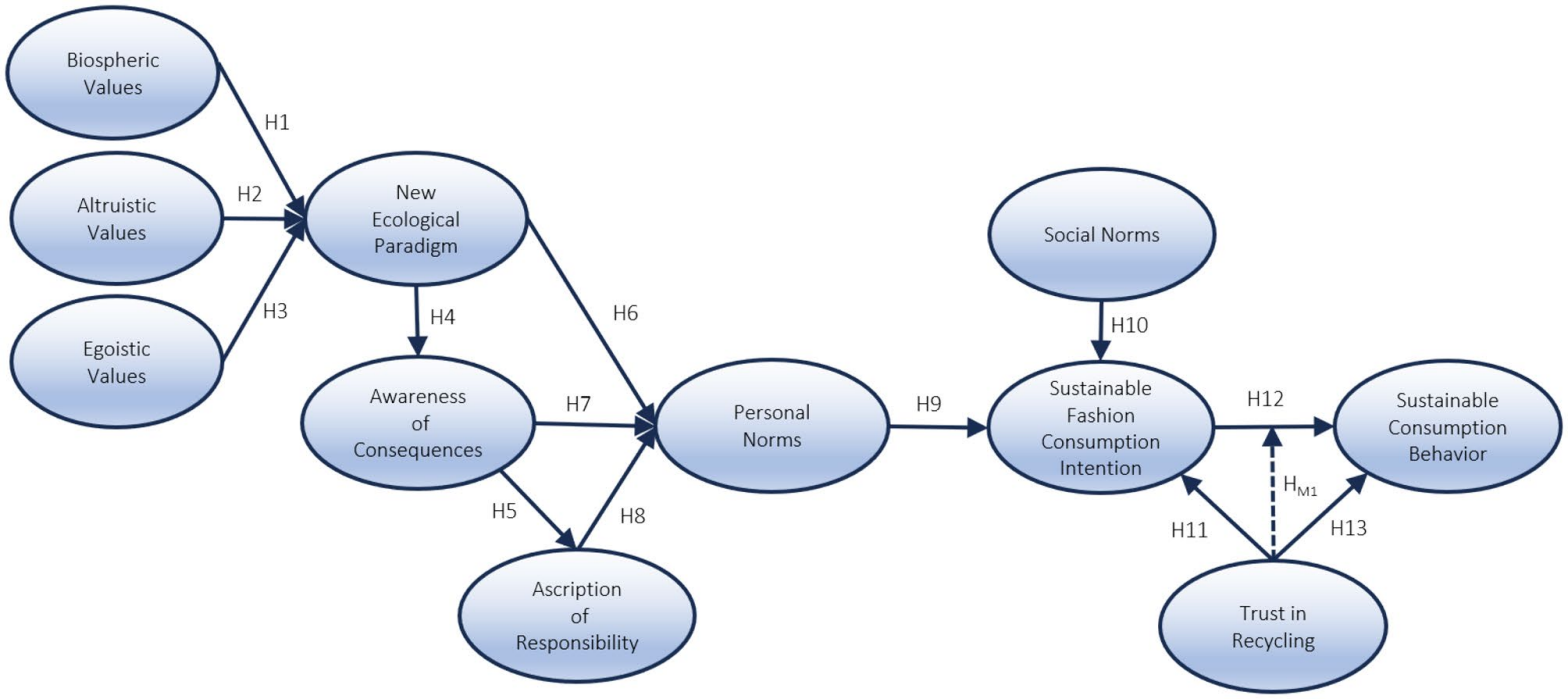
Conceptual framework.

The VBN theory predicts sustainable behaviors in various settings. It has been extensively applied to explain pro-environmental behaviors in various contexts such as residential energy savings^49^, sustainable tourism^50^, climate-conserving behaviors^51^, environmentally friendly cruise^52^, sustainable tourism and hospitality^53^, and reducing air pollution in road transportation^54^. Moreover, the VBN theory has been expanded by the incorporation of supplementary variables, including SN and perceived behavioral control^26^, emotion^55^, satisfaction, trust, and frequency of prior conduct^37^. The initial model encompassed a solitary dimension of norms, specifically personal norms. Kim et al.^56^ argue that in order to achieve a thorough understanding of norms, it is necessary to expand personal norms in conjunction with social norms (SN). This entails considering both internal and exterior norms. The model proposed by Han et al.^37^ received empirical support for trust. In order to address the well-recognized disparity between intentions and behaviors, the current study (Fig. 1) employed trust in recycling (TR) as a variable associated with intentions to engage in sustainable fashion consumption (SCI) and sustainable consumer behavior (SCB). Additionally, the study incorporated SN alongside intrinsic personal norms.

### Hypothesis development

#### Antecedents of new ecological paradigm (EP)

Dunlap et al.^57^ developed the EP, which embraces the idea that humans are an integral part of nature, to explore individuals’ environmental attitudes. Biospheric values (BV) are key factors shaping individuals’ worldviews, particularly in relation to their interest in nature and the environment. The adoption of BV is associated with a greater concern for the environment, emphasizing the central role of environmental values in decision-making and shaping behavior. Similarly, Onel and Mukherjee^23^ found that BV positively impacted a new ecological paradigm. Ünal et al.^58^ explored the relationship between BV and environmental protection, demonstrating that higher levels of BV are associated with increased concern for the environment. Finally, Ye and Tkaczynski^59^ argued that BV is a key driver of engagement with EP, with higher levels of BV corresponding to greater involvement in environmental issues. Therefore, we put forward the following hypothesis:

H_1_. *Biosphere value is positively associated with the new ecological paradigm.*

Altruistic values (AV) refer to a set of ethical principles that prioritize the well-being and interests of others. Altruistic individuals often act in ways that benefit others without expecting anything in return, and may sacrifice their own interests or desires for the greater good. AV are closely linked to the preservation of the social ecology, as noted by Vuorio et al.^60^. By contrast, egoistic value (EV) suggests that environmental issues harm individuals, causing them to prioritize their property, power, and status, and think more about their own resource needs. Previous studies on the correlation between egoistic values and EP have yielded conflicting results. While some studies suggest a negative correlation between EV and EP^61^, Kim’s study on effective hotel environmental management found that only AV were significantly associated with EP, whereas other values did not show significant^62^. In a recent study in Malaysia, Chua et al.^63^ showed that all three values were significant factors of EP, with BV having a medium effect, and EV and AV having small effects. Despite limited robust evidence of the link between AV and EP, considering the cultural and social differences in the samples, it is expected that those values have direct effects on EP. Therefore, the following hypotheses are formulated:

H_2_. *Altruistic value is positively associated with the new ecological paradigm.*

H_3_. *Egoistic value is positively associated with the new ecological paradigm.*

#### Enablers of awareness of consequences (AC)

AC refers to an individual’s understanding of the potential outcomes or effects that may result from their actions or decisions. It involves recognizing the impact of one’s behavior on oneself, others, and the environment. In the context of environmental issues, AC relates to an individual’s understanding of how their actions may affect the natural world and the ecosystems that sustain it. Previous studies have established a causal relationship between EP and AC, indicating that individuals with greater knowledge of current environmental issues tend to be more aware of the impacts of their actions and behaviors. Campos-Soria et al.^64^ and Liobikien and Poškus^65^ supported this correlation, highlighting its positive effect on individuals’ awareness of the consequences of their actions. Han et al.^66^ suggested that an EP can increase people’s awareness of the impact of their actions, while Landon et al.^50^ proposed that personal responsibility can be improved by promoting awareness of this new environmental perspective. Thus, we propose the following hypothesis:

H_4_. *New ecological paradigm is positively associated with the awareness of the consequences.*

#### Enablers of ascription of responsibility (AR)

AR refers to assigning or attributing responsibility for an action or outcome to a particular person or group. This can include both the individual responsibility of a person for their actions, and the responsibility of larger groups or institutions for their impact on society and the environment. According to Ogiemwonyi et al.^67^ &Yang et al.^68^, individuals perceive a feeling of involvement in the preservation of the environment and hold the conviction that they can actively contribute to environmental well-being through the adoption of sustainable practices. When people understand how their actions negatively affect the environment, and take steps to minimize or mitigate that impact, they demonstrate a sense of responsibility associated with personal norms (PN) and AR, as noted by Landon and Boley^50^ and Ghazali et al.^69^. Scholars^58,70^ confirmed this relationship, suggesting that awareness of the consequences of one’s actions positively influences AR. These findings are further supported by Ghazali et al.^69^, who found that awareness of consequences improves the sense of responsibility among both Malaysian and Chinese individuals. Thus, it can be concluded that a greater awareness of the environmental impact of one’s behavior and actions can lead to a stronger sense of responsibility. Thus, we postulated the following hypothesis:

H_5_*. Awareness of the consequences is positively associated with AR.*

#### Antecedents of personal norms

PN refers to internalized beliefs and expectations about how one should behave in a given situation. These norms are self-regulatory in nature and are often shaped by personal values and moral standards. They influence behavior by creating a sense of obligation or duty to act in a certain way, even when external pressures or incentives are absent. PN is thought to be particularly important in the context of pro-environmental behaviors, as it can motivate individuals to act in ways that align with their environmental values, even when it may not be socially or economically advantageous to do so. According to the VBN theory, beliefs are directly linked to individual norms. Thus, people who hold their usual beliefs about ecological well-being are more likely to develop PN for pro-environmental behavior^71^. Using the VBN framework, previous research explored the direct association between EP and PN. Chua et al.^63^ observed the positive effect of EP on PN in a sample of paddy farmers. Similarly, Yeboah and Kaplowitz^72^ found a positive and significant effect of EP on PN among students, teachers, and employees at Michigan State University. Hence, the hypothesis is as follows:

H_6_. *New ecological paradigm is positively associated with the personal norms.*

Individuals who are aware of the negative consequences of not performing altruistic acts have a stronger sense of moral obligation, and are more likely to activate PN to engage in such behaviors. AC is responsible for PN^73^. Understanding the impact of one’s actions on the environment can help individuals take steps to reduce their negative impacts and promote a sense of responsibility, ultimately leading to an increase in PN^50,67^. Gkargkavouzi et al.^74^ suggested that AC effectively promotes personal norms to take necessary action to safeguard the climate. Similarly, Zhang et al.^22^ stated that AC significantly influenced PN engagement in environment-friendly farming practices. Therefore, the following hypothesis is proposed:

H_7_. *Awareness of the consequences is positively associated with PN.*

When individuals experience a stronger sense of personal responsibility, they feel a moral obligation to act. AR refers to assigning responsibility to one’s behavior or actions^69^. Studies show that ascribing responsibility leads to the development of personal norms^75^. If a person recognizes that they have done something wrong, they feel a greater moral obligation to stop or reduce the harm caused^69^. Pro-environmental studies have supported this hypothesis. For instance, Ünal et al.^58^ found that individuals who recognize their ability to reduce the negative consequences of their actions feel a moral obligation to support that behavior. Similarly, Bronfman et al.^70^ and Rezvani et al.^76^ argued that greater AR increases PN. Recent studies^77,78^ on conservation behavior in organizations have demonstrated that AR is the strongest predictor of PN. The hypothesis is as follows:

H_8_. *AR is positively associated with PN.*

#### Enablers of sustainable fashion consumption intention

Schwartz^79^ defines PN as the extent to which an individual feels morally obliged to perform a particular action. Sia and Jose^80^ contend that PN is a key driver of norm-driven pro-environmental behavior and that a stronger sense of personal moral norms can lead to greater engagement in environment-friendly behavior. Han^81^ and Yang et al.^77^ found that PN predicts intentions for environmentally responsible behavior in various groups. Additionally, Ünal et al.^58^ suggest that individuals' intentions to engage in eco-friendly actions increase when they feel a strong sense of obligation. This argument implies that PN leads to planned, environmentally conscious behaviors. Hwang et al.^82^ also revealed that moral obligation has a favorable and substantial effect on the purchase intentions of organic, fair trade, and recycled clothing products. With growing concerns about environmental damage and social inequality in the fashion industry, consumers' personal values have shifted from being self-centered to society-centered^83^. Joanes^16^ discovered a positive and substantial relationship between PN and the intention to minimize personal clothing consumption. Therefore, the following hypothesis is proposed:

H_9_. PN is positively linked with the intention to engage in SCI.

SN refer to shared beliefs about proper behavior within a community regarding one’s response to a situation^84^. Various studies have shown that SN have a positive impact on different behavioral domains related to sustainable behavior^84^. In the context of green consumerism, SN have been found to positively influence consumers’ behavioral intentions to buy green goods and services^26^. Additionally, an individual’s SN have been found to impact their word-of-mouth purchasing intention and intention to sacrifice^52^. Doran and Larsen^85^ found that people are more likely to engage in pro-environmental behavior when they receive messages indicating that people around them are doing the same things. Moreover, Borusiak and Szymkowiak^5^ explained that individuals usually feel pressured to engage in specific behaviors by the people around them. Observing others’ contributions to a common cause, such as environmental preservation, can enhance trust in cooperative intentions, strengthen beliefs about achieving desired outcomes, and increase the willingness to contribute to environmental preservation. Recently, Zahid et al.^30^ revealed a positive relationship between SN and SCI in the case of second-hand clothing in China, while Zhang et al.^29^ found the same relationships in the case of clothing disposal behavior in China. Yeap et al.^27^ found that perceived norms (external influence) have positive influence on the intention to adopt second hand clothes in Malaysian perspectives. Therefore, the following hypothesis is proposed:

H_10_: *There is a positive relationship between SN and SCI.*

Trust is a belief that has been shown to have a positive relationship with green purchasing intention, as established by some authors^86^. Studies indicate that ethical cues can influence consumer opinions about low-performing products^87^, and trust in ethical businesses can influence green buying intentions, while greenwashing can damage consumer trust and reduce their green buying intentions^88^. Thus, companies that want to increase consumers’ green buying intentions should avoid actions that create green skepticism, focus on developing strong relationships with consumers, and build trust in green practices. Therefore, the following hypothesis is proposed:

H_11_: *There is a positive relationship between TR and SCI.*

#### Enfeeblers of Sustainable consumption behavior (SCB)

SCB refers to actions taken by individuals or groups to reduce their negative impacts on the environment and promote sustainable development. This involves making conscious and informed choices when purchasing goods and services, and considering the environmental and social impacts of these choices^89^. Behavioral intention is an important predictor of actual behavior, as people who intend to participate in green behavior are more likely to follow these actions^90^. Gkargkavouzi et al.^74^ revealed that behavioral intention is a significant factor in voluntary actions aimed at mitigating the effects of climate change among Greek respondents. Additionally, taking responsibility for reducing energy consumption in households has been shown to promote energy conservation behaviors in households^91^. The growing need for green products and services suggests that individuals are increasingly adopting environmentally conscious behaviors^92^. Therefore, we hypothesize that as PN supporting green behavior strengthens, individuals are more likely to participate in environmentally conscious actions.

H_12_: *SCI is significantly and positively linked with SCB.*

Green trust refers to individuals’ “willingness to depend on a product or service based on the belief or expectations resulting from its credibility, benevolence, and environmental performance”^93^. A lack of trust can create skepticism, which may negatively affect purchase intention^94^. While most studies have focused on trust as an antecedent of purchase intention^95^, only a few have considered it as a predictor of purchase behavior. Nuttavuthisit and Thøgersen^96^ found that green trust influences green consumption, and Taufique et al.^97^ observed that consumers’ trust in green products leads to pro-environmental consumer behavior. Several recent studies^98–100^ have investigated consumer behavior towards various green products and services. Thus, the following hypothesis is postulated:

H_13_: *TR is positively linked with the SCB.*

#### Moderation of TR

Trust, which refers to consumer beliefs and expectations regarding the reliability, capability, and goodwill of both green products and their producers, leads to the intention to trust the companies and products involved^101^. Individuals pursue sustainable lifestyles not only because of their ecological awareness and comprehension, but also the personal benefits or contentment derived from the products or services they consume^101^. In this study, TR is indicated for second-hand clothes based on its credibility in meeting expectations, usability of clothes, and hygiene issues. Many people want to wear clothes for long periods. Therefore, consumers may feel a lack of trust in the durability of fashion products, as they have already been used for a while. According to Harris and Hagger^102^, the intention to act does not necessarily mean being able to do so. Studies show that consumers’ lack of trust can be a major barrier to purchasing organic products^96^. According to Sultan et al.^103^ and Zheng et al.^104^, trust plays a moderating role in addressing intention-behavior gaps. Therefore, we propose the following hypothesis:

H_M1_: *TR positively moderates the link between SCI and SCB.*

All associations hypothesized above are presented in Fig. 1 below:

## Materials and methods

### Research design

The research followed a cross-sectional design in which an online survey was conducted. This approach differs from that of a longitudinal study that collects data repeatedly within a specific timeframe. This study is quantitative and utilizes a pre-existing research framework. The following sections delve more deeply into the techniques employed in this study.

### Population and sample

The target population was Chinese adults who were more than 18 years old and could participate in the survey without their guardians’ permission. We obtained signed informed consent forms through a questionnaire. Using the G-power 3.1 tool with 10 different predictors, a power of 0.80 and an effect size of 0.15 were utilized to arrive at an estimate for the sample size. The minimum number of samples required to perform an analysis with sufficient power was 118^105^. However, Hair et al.^106^ recommend that partial least squares structural equation modelling (PLS-SEM) should use at least 200 samples. To avoid restrictions of a small sample size, this study intended to obtain data from more than 300 Chinese adults.

### Data collection procedure

Present research created an electronic questionnaire to collect empirical data by applying measurement scale items from previous studies. In addition, this study used a back-translation technique to ensure that the survey was accurate. First, the research questionnaires were carefully examined using English. Second, investigators obtained help from professional experts who were used to the research and spoke both English and Chinese to translate it into Chinese. Third, two professional translators who spoke English and Chinese blindly translated the Chinese questionnaire back into English. Fourth, the quality of the translations was assessed by comparing the two versions. In the case of dispersion, researchers and translators worked to find solutions. Finally, the questionnaire was pretested to determine its accuracy. The issuance and completion of the questionnaires were closely supervised to ascertain the validity and well-organized gathering of the data. Researchers sent 32 questionnaires and collected them from a pretest sample. The pre-test findings confirmed the preliminary validity and reliability of the items used.

The survey collecting data took place in China, since the participants of the research were from different parts of China; the empirical data was obtained using the online survey method. The human research ethics committee of Nanfang College Guangzhou approved this study (Nanfang-2023–1209). This study has been performed in accordance with the Declaration of Helsinki. Written informed consent for participation was obtained from respondents who participated in the survey. Respondents were selected using convenience sampling method. All respondents were informed that the study focused on their sustainable fashion consumption (buying, swapping, or donating second hand clothes). Data was collected between 20^th^ November 2022 to 27^th^ January 2023, and downloaded from the website 28th^th^ January 2023. The questionnaire was circulated using social media platforms, primarily through the WJX.cn website, and 979 valid responses were received.

### Measurement instruments

The questionnaire consisted of two main sections: A and B. Section A comprised the demographic information of respondents (eight questions), whereas all related 54 questions were incorporated in section B. The study used pre-literature for its scale after fitting it into the context, with modification (rewording) and alternation, where necessary. Four items of BV and AV and five items of EV were extracted from Han et al.^107^. Five items each for the contracts SN, TR, and SCB were adopted from Kim et al.^108^, Chen^93^, and Attiq et al.^109^, respectively. The EP construct (five items) was adapted from López-Mosquera and Sánchez^110^, while the five items of AC originated from López-Mosquera and Sánchez^110^ and Choi et al.^111^. The study followed López-Mosquera and Sánchez^110^ and Ünal et al.^58^ for taking the items (5) of AR. The items (5) of PN were sourced from Choi et al.^111^ and Ünal et al.^58^. The TR (five items) was obtained from Chen^93^, and the SCI constructs (five items) were extracted from^5^. The questionnaire had closed-ended items that evaluated 11 constructs from previous studies using a seven-point Likert scale ranging from 1: strongly disagree to 7: strongly agree. A complete questionnaire has been submitted to this manuscript as supporting material—*S1. Survey Instrument.*

### Common method bias

To determine the impact of common method bias (CMB) and propose remedies, the study questions were meticulously crafted, and respondents were assured that there were no right or wrong answers, and their responses would remain anonymous^112^. Harman’s single-factor test was used as a diagnostic tool to determine the influence of common method bias. The single factor was 32.507%, which was less than the prescribed limit of 50% in Harman’s one-factor test. This proves that the CMB had no significant effect on this study. Moreover, an examination of CMB involved an assessment of the full collinearity of all constructs, as recommended by Kock^113^. All the study structures were regressed on the common variance and variance inflation factors (VIF) values shown in Table 1. There was no presence of bias in the data from a single source because all VIF values were lower than 3.3.

**Table 1:** Full collinearity test.

	BV	AV	EV	EP	AC	AR	PN	SN	GR	SCI	SCB
VIF values	1.360	1.455	1.419	1.284	1.435	1.512	1.533	1.365	1.774	1.952	2.073

*BV* biospheric values, *AV* altruistic values, *EV* egoistic values, *EP* new ecological paradigm, *AC* awareness of consequences, *AR* ascription of responsibility, *PN* personal norms, *SN* social norms, *TR* trust in recycling, *SCI* sustainable fashion consumption intention, *SCB* sustainable fashion consumption behavior, *VIF* variance inflation factors.

### Multivariate normality

Using appropriate data analysis techniques to check multivariate normality is crucial. This study estimated multivariate normality using an online Web Power tool^114^. The results of the multivariate normality test revealed that the p values for Mardia’s multivariate skewness (z = 2209.93) and kurtosis (z = 37.85) were below 0.05, indicating non-normality. Thus, to accommodate non-normal data, this study uses PLS-SEM. Structural equation modelling approach provides better estimates than regression for mediation and moderation^115^. PLS-SEM is a satisfactory approach for evaluating complex frameworks involving moderating relationships^106^. Therefore, PLS-SEM was employed with the Smart-PLS 4.0.

### Data analysis method

The analysis of this research followed two stages. First, the measurement model was quantified to determine validity and reliability. In the later phase, structural equation modeling was used to elaborate the connection between the predictor and latent variables, including the mediation and moderation effects. It is widely accepted that structural equation modeling provides better estimates than regression when executing mediation and moderation^115^. Therefore, this research applied structural equation modeling, specifically PLS-SEM, using Smart-PLS 4.0, which is regarded as the best choice because of its effectiveness in evaluating complex frameworks involving moderation effects^106^.

### Ethics approval

The human research ethics committee of Nanfang College Guangzhou approved this study (Nanfang-2022-1009). This study has been performed in accordance with the Declaration of Helsinki.

### Informed consent

Written informed consent for participation was obtained from respondents who participated in the survey.

## Findings

### Respondents profile

The provided demographic Table 2 presents information on the gender, age group, education level, marital status, employment status, and clothing purchasing habits of 979 individuals. The sample was evenly divided into male (49.7%) and female (50.3%) participants. Most participants had a bachelor’s degree or below (93.0%), with only 11.1% having a postgraduate degree. In terms of age, the sample was evenly distributed across different age groups, with the largest groups being those aged 26–35 years (28.1%) and 36–45 years (29.3%). Most participants were married (61.5%) and employed full-time (41.3%), with 13.2% unemployed and 3.7% retired. Regarding clothing purchasing habits, most participants purchased new clothes one to two times per month (34.8%), and spent less than RMB1500 per month on clothing (39.7%).

**Table 2:** Demographic characteristics.

	N	%		N	%
Gender			Education		
Male	487	49.7	Diploma/advanced diploma	429	43.8
Female	492	50.3	Bachelor	373	38.1
Total	979	100.0	Postgraduate degree	109	11.1
			Others	68	6.9
*Age group*			Total	979	100.0
18–25 years	190	19.4			
26–35 years	275	28.1	Marital status		
36–45 years	287	29.3	Single	298	30.4
46–55 years	121	12.4	Married	602	61.5
56–65 years	69	7.0	Divorced	60	6.1
Above 65 years	37	3.8	Widow	19	1.9
Total	979	100.0	Total	979	100.0
Employment status			Average monthly income		
Employed full time	404	41.3	Less than RMB1500	247	25.2
Employed part time	188	19.2	RMB1500–RMB3000	133	13.6
Self-employed	141	14.4	RMB3001–RMB4500	240	24.5
Student	81	8.3	RM4501–RMB6000	208	21.2
Unemployed	129	13.2	RMB6001–RMB7500	102	10.4
Retired	36	3.7	More than RMB 7500	49	5.0
Total	979	100.0	Total	979	100.0
How often do you purchase new cloths?			How much do you spend on new clothing per month?		
Seldom	299	30.5	Less than RMB1500	389	39.7
One to two times a month	341	34.8	RMB1500–RMB3000	117	12.0
Three to five times a month	194	19.8	RMB3001–RMB4500	205	20.9
Six to ten times a month	118	12.1	RM4501–RMB6000	150	15.3
Almost every day	27	2.8	RMB6001–RMB7500	93	9.5
Total	979	100.0	More than RMB 7500	25	2.6
			Total	979	100.0

1 USD = 6.35 CNY.

The study’s reliability and validity were examined by assessing the internal consistency of the measurement items as well as the composite reliability and average variance extracted (Table 3). The results indicate high levels of internal consistency, with Cronbach’s alpha ranging from 0.892 to 0.933 for each variable. The composite reliability values ranged from 0.896 to 0.947, indicating a high degree of construct reliability. The average variance extracted values ranged from 0.711 to 0.788, indicating that the measures accounted for a substantial proportion of the variance in each construct. The variance inflation factor values were all below the recommended threshold of 2.5, indicating no multicollinearity issues. Therefore, the measures demonstrated good reliability and validity, suggesting that the results are credible and robust.

**Table 3:** Reliability and validity.

Variables	Items	Mean	Std. deviation	Cronbach's alpha	Composite reliability (rho_a)	Composite reliability (rho_c)	Average variance extracted	Variance inflation factors
BV	4	4.708	1.613	0.911	0.911	0.937	0.788	1.233
AV	4	4.637	1.599	0.892	0.896	0.925	0.755	1.231
EV	5	4.743	1.420	0.913	0.915	0.935	0.742	1.176
EP	5	4.725	1.476	0.899	0.902	0.925	0.711	1.125
AC	6	4.840	1.412	0.933	0.937	0.947	0.748	1.174
AR	5	5.034	1.369	0.905	0.908	0.929	0.725	1.185
PN	5	4.968	1.480	0.920	0.922	0.940	0.757	1.314
SN	5	4.901	1.427	0.918	0.918	0.938	0.753	1.211
TR	5	4.798	1.399	0.906	0.907	0.930	0.726	1.485
SCI	5	4.852	1.402	0.909	0.912	0.932	0.734	1.504
SCB	5	4.975	1.401	0.910	0.911	0.933	0.736	-

*BV* biospheric values, *AV* altruistic values, *EV* egoistic values, *EP* new ecological paradigm, *AC* awareness of consequences, *AR* ascription of responsibility, *PN* personal norms, *SN* social norms, *TR* trust in recycling, *SCI* sustainable fashion consumption intention, *SCB* sustainable fashion consumption behavior, *VIF* variance inflation factors.

**Figure 2:**
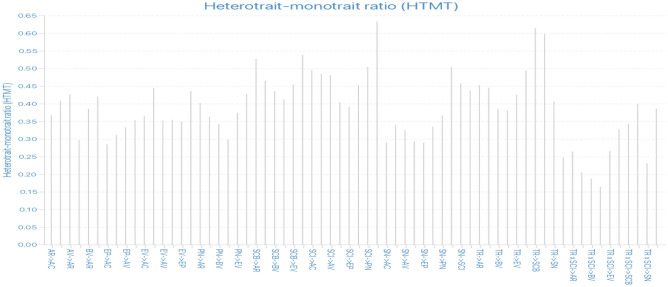
Heterotrait–monotrait ratio (HTMT) matrix.

After confirming reliability and discriminant validity, we applied both Fornell and Lacker’s criterion and Heterotrait-Monotrait (HTMT) ratio (Table 4; Fig. 2). The Fornell–Larcker criterion shows the correlation between constructs and the amount of variance shared among them. Diagonal values represent the square root of the average variance extracted (AVE) for each construct. Values above the diagonal represent the correlation between constructs, whereas those below the diagonal represent the AVE for each construct. All diagonal values exceed 0.5, indicating acceptable convergent validity. The off-diagonal values were generally lower, suggesting good discriminant validity. The standard value of the HTMT is less than 0.90, and values exceeding this limit indicate low discriminant validity^116^. All values in the HTMT matrix are below the threshold value (i.e., 0.90) confirming a high level of discriminant validity. However, all cross-loadings remained greater than 0.5, as shown (Appendix 1). Overall, the analysis suggests that the measures have adequate reliability and validity for the constructs studied.

**Table 4:** Loading, cross-loading and Fornell–Larcker criterion.

	AC	AR	AV	BV	EP	EV	PN	SCB	SCI	SN	TR
AC1	0.867	0.314	0.318	0.222	0.258	0.294	0.379	0.322	0.390	0.226	0.357
AC2	0.880	0.353	0.337	0.278	0.255	0.355	0.389	0.407	0.421	0.281	0.387
AC3	0.861	0.258	0.324	0.236	0.206	0.282	0.353	0.322	0.403	0.220	0.342
AC4	0.863	0.268	0.322	0.236	0.215	0.258	0.350	0.319	0.399	0.203	0.327
AC5	0.863	0.302	0.308	0.220	0.235	0.283	0.348	0.347	0.403	0.239	0.334
AC6	0.854	0.264	0.331	0.228	0.197	0.273	0.280	0.328	0.358	0.223	0.338
AR1	0.245	0.825	0.330	0.292	0.217	0.332	0.301	0.410	0.371	0.281	0.331
AR2	0.284	0.864	0.315	0.288	0.243	0.361	0.305	0.394	0.364	0.271	0.346
AR3	0.322	0.868	0.357	0.314	0.263	0.342	0.341	0.417	0.360	0.267	0.347
AR4	0.311	0.859	0.311	0.299	0.222	0.354	0.305	0.408	0.406	0.270	0.372
AR5	0.289	0.840	0.321	0.297	0.248	0.335	0.314	0.408	0.383	0.232	0.351
AV1	0.368	0.361	0.872	0.337	0.288	0.319	0.319	0.367	0.409	0.263	0.366
AV2	0.329	0.369	0.855	0.351	0.245	0.254	0.306	0.387	0.379	0.286	0.383
AV3	0.290	0.313	0.882	0.311	0.264	0.270	0.265	0.360	0.366	0.222	0.308
AV4	0.307	0.290	0.867	0.321	0.247	0.265	0.257	0.342	0.358	0.250	0.337
BV1	0.230	0.301	0.338	0.884	0.291	0.297	0.284	0.355	0.333	0.270	0.332
BV2	0.241	0.322	0.350	0.891	0.293	0.282	0.278	0.346	0.320	0.218	0.289
BV3	0.264	0.314	0.353	0.891	0.277	0.296	0.288	0.345	0.323	0.237	0.306
BV4	0.241	0.306	0.306	0.886	0.276	0.269	0.267	0.361	0.333	0.228	0.315
EP1	0.271	0.230	0.289	0.262	0.856	0.301	0.267	0.327	0.311	0.254	0.303
EP2	0.222	0.246	0.279	0.297	0.844	0.270	0.219	0.334	0.311	0.205	0.324
EP3	0.180	0.222	0.225	0.259	0.842	0.274	0.222	0.314	0.271	0.213	0.281
EP4	0.216	0.279	0.213	0.266	0.839	0.255	0.228	0.305	0.309	0.230	0.294
EP5	0.222	0.210	0.258	0.267	0.837	0.240	0.213	0.294	0.289	0.207	0.252
EV1	0.282	0.386	0.275	0.273	0.273	0.864	0.291	0.362	0.331	0.252	0.322
EV2	0.284	0.378	0.302	0.303	0.274	0.868	0.330	0.399	0.406	0.286	0.347
EV3	0.312	0.333	0.243	0.243	0.293	0.883	0.287	0.339	0.355	0.249	0.341
EV4	0.299	0.350	0.312	0.303	0.282	0.858	0.294	0.357	0.378	0.302	0.333
EV5	0.280	0.295	0.247	0.268	0.246	0.832	0.280	0.326	0.310	0.232	0.326
PN1	0.351	0.338	0.274	0.268	0.240	0.291	0.885	0.427	0.398	0.277	0.393
PN2	0.355	0.353	0.295	0.298	0.273	0.321	0.879	0.456	0.406	0.334	0.407
PN3	0.371	0.283	0.278	0.282	0.235	0.271	0.868	0.435	0.397	0.281	0.377
PN4	0.371	0.340	0.327	0.268	0.228	0.327	0.863	0.439	0.440	0.314	0.410
PN5	0.323	0.284	0.260	0.249	0.211	0.283	0.856	0.388	0.370	0.261	0.383
SCB1	0.358	0.414	0.379	0.364	0.343	0.353	0.433	0.835	0.481	0.402	0.454
SCB2	0.336	0.427	0.334	0.333	0.289	0.338	0.439	0.874	0.521	0.409	0.507
SCB3	0.341	0.390	0.379	0.330	0.310	0.361	0.392	0.860	0.460	0.400	0.482
SCB4	0.343	0.414	0.358	0.345	0.347	0.370	0.425	0.875	0.541	0.413	0.471
SCB5	0.322	0.406	0.349	0.327	0.317	0.356	0.429	0.844	0.474	0.354	0.484
SCI1	0.407	0.416	0.395	0.342	0.288	0.388	0.419	0.533	0.859	0.407	0.497
SCI2	0.411	0.400	0.393	0.315	0.296	0.339	0.394	0.515	0.870	0.351	0.488
SCI3	0.390	0.371	0.360	0.320	0.332	0.367	0.405	0.501	0.861	0.356	0.456
SCI4	0.391	0.396	0.374	0.305	0.300	0.369	0.397	0.489	0.863	0.351	0.455
SCI5	0.365	0.299	0.339	0.293	0.303	0.306	0.367	0.434	0.829	0.326	0.431
SN1	0.230	0.266	0.268	0.259	0.229	0.266	0.301	0.381	0.364	0.855	0.316
SN2	0.237	0.287	0.268	0.226	0.235	0.284	0.328	0.431	0.376	0.880	0.338
SN3	0.251	0.265	0.242	0.228	0.214	0.244	0.289	0.401	0.367	0.875	0.318
SN4	0.219	0.265	0.246	0.228	0.219	0.266	0.281	0.410	0.357	0.868	0.308
SN5	0.236	0.260	0.248	0.224	0.249	0.272	0.267	0.377	0.355	0.860	0.329
TR1	0.344	0.333	0.328	0.284	0.292	0.305	0.383	0.482	0.471	0.294	0.850
TR2	0.356	0.363	0.353	0.309	0.294	0.372	0.393	0.495	0.495	0.344	0.853
TR3	0.344	0.375	0.360	0.312	0.283	0.326	0.386	0.492	0.455	0.302	0.864
TR4	0.341	0.347	0.346	0.297	0.310	0.324	0.405	0.477	0.462	0.310	0.855
TR5	0.333	0.329	0.318	0.287	0.295	0.322	0.362	0.434	0.433	0.331	0.838
AC	0.865										
AR	0.342	0.851									
AV	0.374	0.384	0.869								
BV	0.275	0.350	0.379	0.888							
EP	0.265	0.281	0.302	0.320	0.843						
EV	0.339	0.405	0.320	0.322	0.319	0.861					
PN	0.408	0.369	0.331	0.314	0.273	0.344	0.870				
SCB	0.396	0.479	0.419	0.396	0.374	0.414	0.494	0.858			
SCI	0.459	0.442	0.436	0.369	0.354	0.414	0.464	0.579	0.857		
SN	0.270	0.310	0.293	0.268	0.264	0.307	0.338	0.461	0.420	0.868	
TR	0.404	0.411	0.401	0.350	0.346	0.387	0.453	0.559	0.545	0.371	0.852

The bold off-diagonal values are the square root of AVE.

*BV* biospheric values, *GT* green trust, *AV* altruistic values, *PN* personal norms, *EV* egoistic values, *AC* awareness of consequences, *EP* new ecological paradigm, *IGP* intention towards green consumption practices, *AR* ascription of responsibility, *AGP* adoption of green consumption practices.

**Figure 3:**
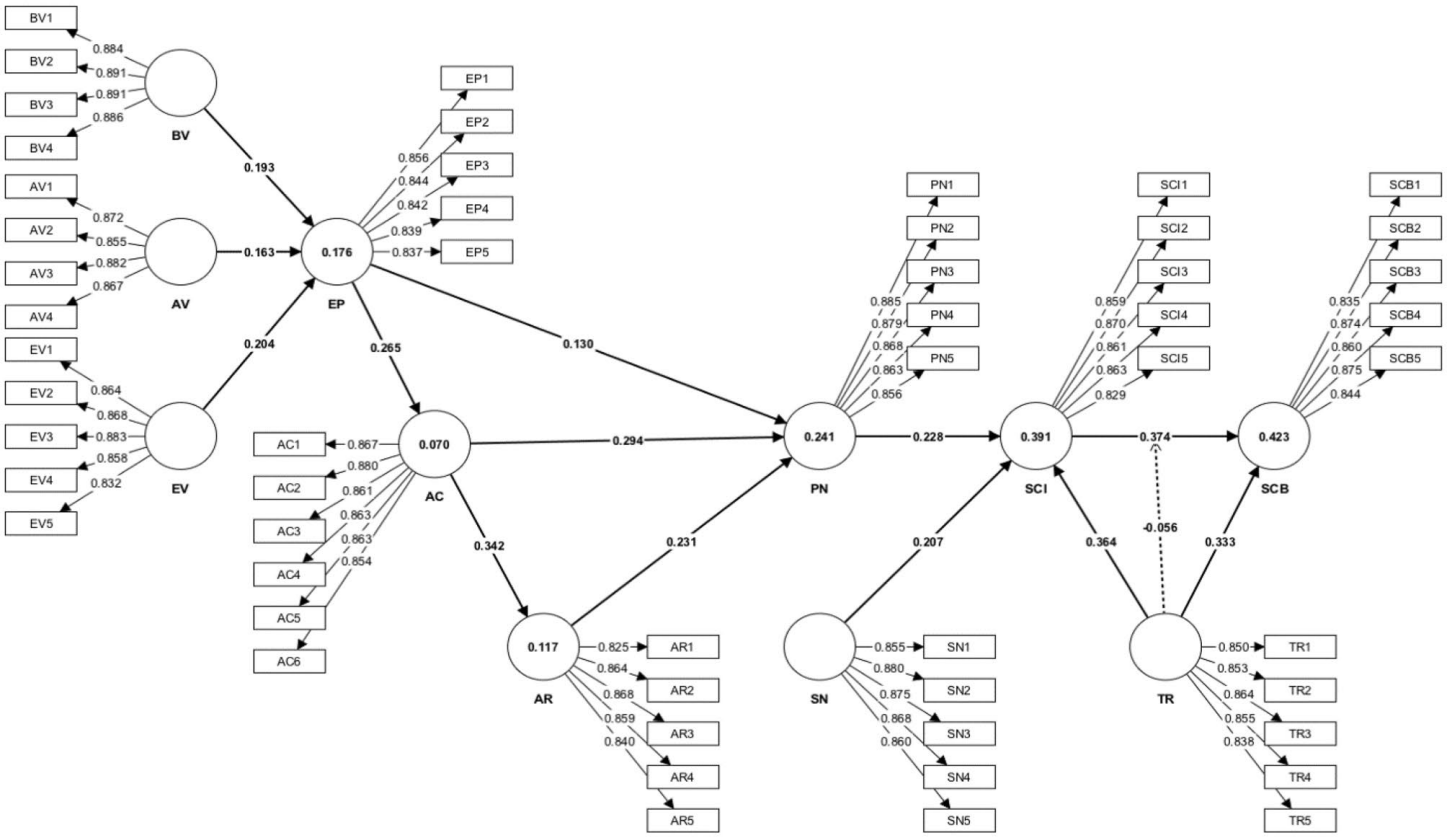
Measurement model.

**Figure 4:**
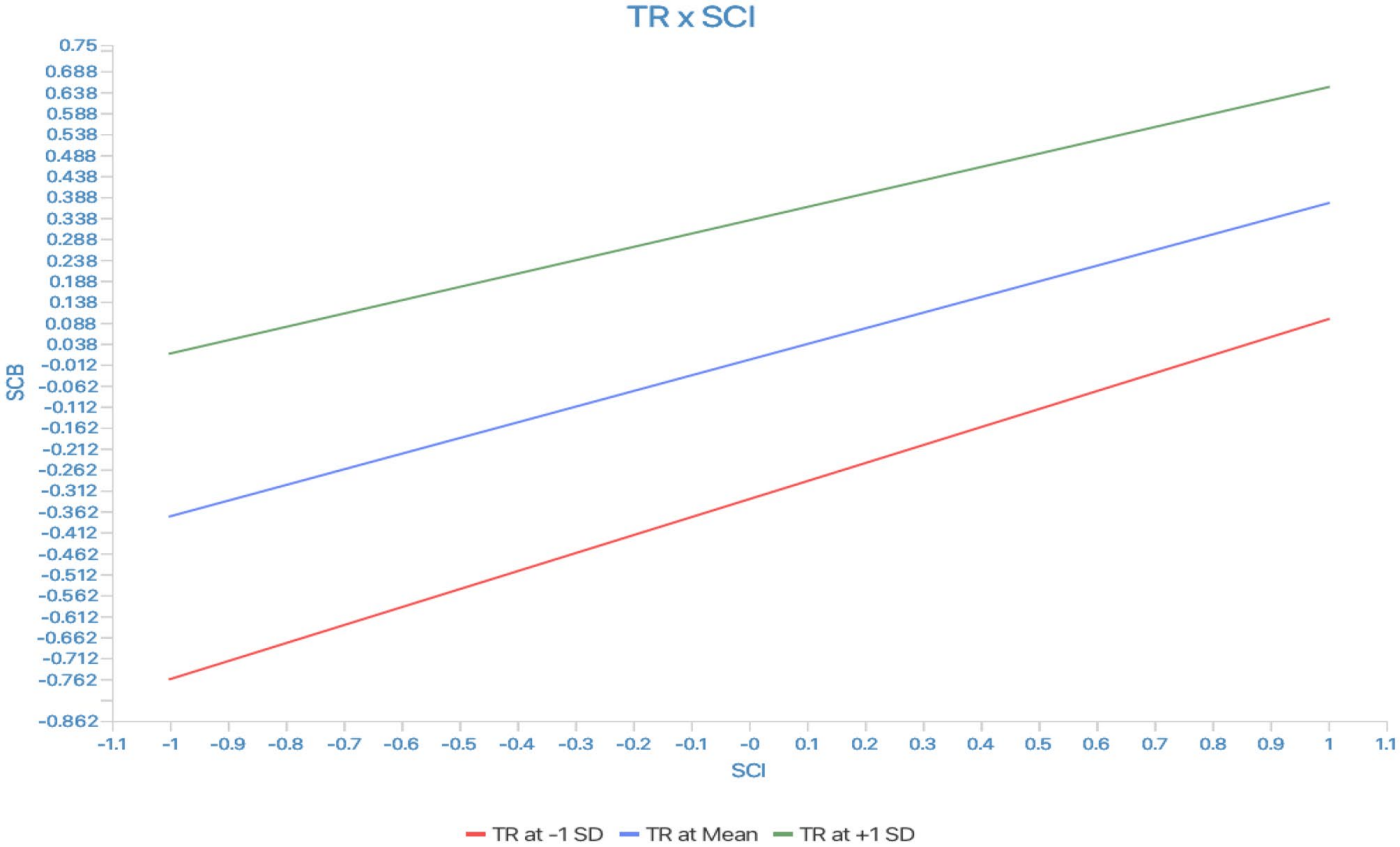
Moderation of trust in recycling.

The results (as presented in Table 5; Fig. 3) reveal that BV (β = 0.193, t = 5.091, p < 0.01), AV (β = 0.163, t = 4.378, p < 0.01) and EV (β = 0.204, t = 5.608, p < 0.01) influenced significantly on EP. The link between EP and AC was identified as positive (β = 0.265, t = 7.998, p < 0.01), signifying the positive effect of EP on AC. Moreover, AC had a positive significant influence on AR (β = 0.342, t = 10.591, p < 0.01). EP (β = 0.130, t = 3.712, p < 0.01), AC (β = 0.294, t = 7.963, p < 0.01) and AR (β = 0.231, t = 6.530, p < 0.01) affected positively on PN. Additionally, PN (β = 0.228, t = 6.645, p < 0.01), SN (β = 0.207, t = 6.170, p < 0.01) and TR (β = 0.364, t = 10.777, p < 0.01) demonstrated a positive relation on SCI. SCI (β = 0.374, t = 11.140, p < 0.01) and TR (β = 0.333, t = 9.759, p < 0.01) had significant positive effects on SCB. Similarly, the study finds a statistically insignificant moderation role of TR (β = , t = − 0.056, t = 2.071, p < 0.05) within the connection between SCI and SCB (Fig. 4). Although the p value (0.019) falls within the 5% level of significance, the hypothesis is rejected as it presents the opposite relationship. Therefore, this study found that hypotheses (H1–13) were validated at the 1% level of significance, and hypothesis (H_M1_) was rejected (Table 5).

**Table 5:** Hypothesis testing.

Hypothesis		Beta	CI Min	CI Max	Std. error	*t *value	*p *value	*f*^2^	*r*^2^	*Q*^2^	*Decision*
H_1_	BV → EP	0.193	0.131	0.254	0.038	5.091**	0.000	0.037	0.176	0.123	Accepted
H_2_	AV → EP	0.163	0.101	0.224	0.037	4.378**	0.000	0.026			Accepted
H_3_	EV → EP	0.204	0.144	0.265	0.036	5.608**	0.000	0.043			Accepted
H_4_	EP → AC	0.265	0.212	0.321	0.033	7.998**	0.000	0.076	0.070	0.051	Accepted
H_5_	AC → AR	0.342	0.290	0.396	0.032	10.591**	0.000	0.133	0.117	0.084	Accepted
H_6_	EP → PN	0.130	0.072	0.187	0.035	3.712**	0.000	0.020	0.241	0.179	Accepted
H_7_	AC → PN	0.294	0.233	0.354	0.037	7.963**	0.000	0.097			Accepted
H_8_	AR → PN	0.231	0.174	0.290	0.035	6.530**	0.000	0.060			Accepted
H_9_	PN → SCI	0.228	0.172	0.284	0.034	6.645**	0.000	0.065	0.391	0.284	Accepted
H_10_	SN → SCI	0.207	0.152	0.263	0.034	6.170**	0.000	0.058			Accepted
H_11_	TR → SCI	0.364	0.307	0.418	0.034	10.777**	0.000	0.162			Accepted
H_12_	SCI → SCB	0.374	0.317	0.427	0.034	11.140**	0.000	0.161	0.423	0.308	Accepted
H_13_	TR → SCB	0.333	0.276	0.389	0.034	9.759**	0.000	0.129			Accepted
H_M1_	TR*SCI → SCB	− 0.056	− 0.102	− 0.012	0.027	2.071*^	0.019	0.006			Rejected*

*BV* biospheric values, *AV* altruistic values, *EV* egoistic values, *EP* new ecological paradigm, *AC* awareness of consequences, *AR* ascription of responsibility, *PN* personal norms, *SN* social norms, *GR* trust in recycling, *SCI* sustainable fashion consumption intention, *SCB* sustainable fashion consumption behavior.

**Significant at 1% level of significance, &,*indicates significant at 5% level of significance. ^The hypothesis is rejected due to its opposite result as hypothesized.

Moreover, the outcome (Table 6) of the study found that BV, AV, and EV had indirect relationships with AC and PN, and EP has the same with AR, PN, and SCI. Likewise, AC is indirectly linked with PN and SCI, while AR is with SCI. The constructs PN, SN, and TR are indirectly related on the SCB.

**Table 6:** Specific indirect effects.

	Beta	CI Min	CI Max	Std. error	*t *value	*p* value
BV → EP → AC	0.051	0.033	0.072	0.012	4.227	0.000
BV → EP → AC → AR	0.018	0.011	0.026	0.005	3.725	0.000
BV → EP → AC → AR → PN	0.004	0.002	0.006	0.001	3.186	0.001
BV → EP → AC → AR → PN → SCI	0.001	0.000	0.002	0.000	2.728	0.003
BV → EP → AC → AR → PN → SCI → SCB	0.000	0.000	0.001	0.000	2.520	0.006
BV → EP → AC → PN	0.015	0.009	0.022	0.004	3.737	0.000
BV → EP → AC → PN → SCI	0.003	0.002	0.006	0.001	3.069	0.001
BV → EP → AC → PN → SCI → SCB	0.001	0.001	0.002	0.000	2.844	0.002
BV → EP → PN	0.025	0.012	0.040	0.009	2.885	0.002
BV → EP → PN → SCI	0.006	0.003	0.010	0.002	2.563	0.005
BV → EP → PN → SCI → SCB	0.002	0.001	0.004	0.001	2.408	0.008
AV → EP → AC	0.043	0.025	0.065	0.012	3.564	0.000
AV → EP → AC → AR	0.015	0.008	0.023	0.005	3.200	0.001
AV → EP → AC → AR → PN	0.003	0.002	0.006	0.001	2.790	0.003
AV → EP → AC → AR → PN → SCI	0.001	0.000	0.001	0.000	2.448	0.007
AV → EP → AC → AR → PN → SCI → SCB	0.000	0.000	0.001	0.000	2.277	0.011
AV → EP → AC → PN	0.013	0.007	0.020	0.004	3.272	0.001
AV → EP → AC → PN → SCI	0.003	0.001	0.005	0.001	2.761	0.003
AV → EP → AC → PN → SCI → SCB	0.001	0.001	0.002	0.000	2.572	0.005
AV → EP → PN	0.021	0.010	0.034	0.008	2.770	0.003
AV → EP → PN → SCI	0.005	0.002	0.008	0.002	2.486	0.006
AV → EP → PN → SCI → SCB	0.002	0.001	0.003	0.001	2.325	0.010
EV → EP → AC	0.054	0.035	0.076	0.013	4.261	0.000
EV → EP → AC → AR	0.019	0.011	0.027	0.005	3.749	0.000
EV → EP → AC → AR → PN	0.004	0.002	0.007	0.001	3.177	0.001
EV → EP → AC → AR → PN → SCI	0.001	0.001	0.002	0.000	2.785	0.003
EV → EP → AC → AR → PN → SCI → SCB	0.000	0.000	0.001	0.000	2.620	0.004
EV → EP → AC → PN	0.016	0.010	0.023	0.004	3.970	0.000
EV → EP → AC → PN → SCI	0.004	0.002	0.006	0.001	3.284	0.001
EV → EP → AC → PN → SCI → SCB	0.001	0.001	0.002	0.000	3.107	0.001
EV → EP → PN	0.027	0.013	0.043	0.009	2.949	0.002
EV → EP → PN → SCI	0.006	0.003	0.010	0.002	2.647	0.004
EV → EP → PN → SCI → SCB	0.002	0.001	0.004	0.001	2.519	0.006
EP → AC → AR	0.091	0.067	0.119	0.016	5.766	0.000
EP → AC → AR → PN	0.021	0.014	0.030	0.005	4.207	0.000
EP → AC → AR → PN → SCI	0.005	0.003	0.007	0.001	3.363	0.000
EP → AC → AR → PN → SCI → SCB	0.002	0.001	0.003	0.001	3.039	0.001
EP → AC → PN	0.078	0.057	0.101	0.013	5.910	0.000
EP → AC → PN → SCI	0.018	0.011	0.026	0.004	4.091	0.000
EP → AC → PN → SCI → SCB	0.007	0.004	0.010	0.002	3.671	0.000
EP → PN → SCI	0.030	0.015	0.046	0.009	3.145	0.001
EP → PN → SCI → SCB	0.011	0.005	0.018	0.004	2.902	0.002
AC → AR → PN	0.079	0.057	0.104	0.014	5.531	0.000
AC → AR → PN → SCI	0.018	0.011	0.026	0.004	4.042	0.000
AC → AR → PN → SCI → SCB	0.007	0.004	0.010	0.002	3.559	0.000
AC → PN → SCI	0.067	0.045	0.092	0.014	4.761	0.000
AC → PN → SCI → SCB	0.025	0.016	0.036	0.006	4.181	0.000
AR → PN → SCI	0.053	0.035	0.073	0.012	4.511	0.000
AR → PN → SCI → SCB	0.020	0.012	0.029	0.005	3.938	0.000
PN → SCI → SCB	0.085	0.060	0.113	0.016	5.242	0.000
SN → SCI → SCB	0.077	0.054	0.103	0.015	5.083	0.000
TR → SCI → SCB	0.136	0.110	0.162	0.016	8.532	0.000

*BV* biospheric values, *AV* altruistic values, *EV* egoistic values, *EP* new ecological paradigm, *AC* awareness of consequences, *AR* ascription of responsibility, *PN* personal norms, *SN* social norms, *GR* trust in recycling, *SCI* sustainable fashion consumption intention, *SCB* sustainable fashion consumption behavior.

## Discussion

This study investigated the relationship between values, attitudes, and sustainable fashion consumption behavior. It proposed 14 hypotheses based on the extended VBN theory, 13 of which were confirmed by empirical investigation. The exogenous constructs in the model were found to have a significant impact on the endogenous construct, with an explanatory power of 42.3% for SCB, indicating a good fit between the model and the investigation. The following discussion provides details of the relationships identified in this study.

The finding that Biospheric, Altruistic, and Egoistic values had a significant influence on the New Ecological Paradigm (NEP) is consistent with previous research^63^ that highlights the importance of values in shaping environmental attitudes and behavior (H1–3). The finding that these values significantly influence the New Ecological Paradigm suggests that individuals who hold these values are more likely to adopt an environmentalist perspective and engage in sustainable behaviors. The results also suggest that individuals who hold values that prioritize the environment, personal and collective well-being, and personal growth, are more likely to adopt a new ecological paradigm that views humans as part of the ecosystem and emphasizes the importance of protecting the natural environment.

The present study revealed that the New Ecological Paradigm significantly influences individuals’ Awareness of Consequences (H4), indicating that people who hold environmental concerns and beliefs tend to be more aware of the environmental consequences of their actions. These findings are consistent with previous research showing that the New Ecological Paradigm is an essential predictor of individuals’ AC^50^. Han et al.^66^ suggest that individuals who hold a new ecological paradigm perspective are more likely to be aware of the environmental consequences of their actions. This new ecological paradigm represents an underlying belief system that recognizes the interdependence between humans and the natural environment. Therefore, it can be assumed that individuals with a new ecological paradigm perspective are more likely to be conscious of the consequences of their actions on the environment. Consequently, they may be more motivated to engage in SCB to reduce their environmental impact.

Furthermore, the finding that awareness of consequences had a positive and significant influence on AR (H5) supports previous research^58,69^ that has suggested that individuals who are more aware of the consequences of their actions are more likely to feel responsible for their impact on the environment. This finding aligns with Value-Belief-Norm theory, which proposes that individuals who are aware of ecological consequences accept responsibility for their actions. In this study, the relationship between awareness of consequences and AR may be explained by the fact that individuals who are more aware of the consequences of their actions are more likely to feel a sense of responsibility for their impact on the environment. This finding has implications for interventions aimed at promoting sustainable fashion consumption, as it suggests that increasing awareness of the consequences of unsustainable fashion practices may help individuals develop a stronger sense of responsibility for their actions, and motivate them to engage in more sustainable fashion consumption behaviors.

Moreover, this study identified a positive and significant relationship between EP, AC, AR, and PN (H6–8), consistent with previous studies^22,63,77,78^. This implies that individuals who strongly believe in the need for ecological conservation and are aware of the consequences of their actions towards the environment are more likely to develop a sense of responsibility for their actions. They are more likely to form a PN that prioritizes sustainable fashion consumption, which could result in behavioral changes towards more sustainable fashion choices. Individuals who internalize sustainable values and beliefs are more likely to form PN that prioritize sustainable fashion consumption, leading to changes in their behavior. This finding suggests that interventions aimed at promoting sustainable fashion consumption should focus on developing PN that prioritize the environment and sustainability.

Additionally, the results of this study suggest that PN, SN, and TR have positive effects on SCI (H9–11). This is important because it indicates that personal influence, social influence, and TR motivate individuals to adopt SCB. SN refers to shared beliefs and behaviors within a society or a particular group. The positive effect of social norms on SCI implies that individuals are more likely to engage in SCB when they perceive such behaviors as socially accepted and valued. This finding is consistent with previous research^84^_._ Yeap et al.^27^, Zahid et al.^30^, Zhang et al.^22^ demonstrating the important role of SN in shaping pro-environmental behaviors. TR, on the other hand, refers to an individual’s confidence in the effectiveness and efficiency of recycling programs. The positive effect of TR on SCI suggests that individuals who trust recycling programmes are more likely to engage in SCB. This finding is particularly relevant^81^ in the context of sustainable fashion consumption, as it suggests that individuals in TR programs may be more likely to engage in behaviors such as recycling clothes or purchasing clothes made from recycled materials. Also, this is true for renting clothes in case of circular fashion conducted by Shrivastava et al.^117^.

Finally, this study found that SCI and TR were significant predictors of SCB (H12–13). This result suggests that individuals who have a higher intention to consume sustainability and TR are more likely to engage in SCB, such as buying, swapping, or donating second-hand clothes. The positive effect of SCI on SCB suggests that individuals with a strong intention to engage in sustainable fashion consumption are more likely to engage in such behaviors. This finding is consistent with previous research^86^ demonstrating the important role of intention in predicting pro-environmental behaviors. However, the study found that TR did not moderate the relationship between SCI and SCB, contrary to the hypothesized relationship (H_M1_) and the findings of Sultan et al.^103^.

## Implications of the study

### Theoretical implications

This study makes several theoretical contributions to the field of sustainable fashion consumption. *First,* it identifies several factors that influence sustainable fashion consumption behavior, including BV, AV, and EV, as well as AC, AR, PN, SN, and TR. This provides a comprehensive understanding of the factors that motivate individuals to engage in SCB, which can inform the development of interventions aimed at promoting such behaviors. *Second,* it used bi-dimensional norms, such as PN and SN, in the VBN frameworks, and established that both are predictors of SCB. Thus, this study contributes to the literature on the role of SN in promoting pro-environmental behavior. The finding that SN has a positive effect on SCI suggests that interventions aimed at increasing SN to support sustainable fashion consumption may be effective in promoting SCB.

*Third,* this study identified the importance of SCI and TR as predictors of SCB. This highlights the importance of considering not only individuals’ values and beliefs, but also their perceptions of the effectiveness and efficiency of recycling programs in promoting SCB. *Finally,* this study contributes to the literature on the intention-behavior gap in SCB. Many respondents are highly willing to purchase, but ultimately do not purchase pro-environmental products, which is referred to as the intention-behavior gap. Although these findings suggest that SCI and TR are significant predictors of SCB, there is no evidence that TR is a moderator. This result will encourage academia to conduct further research on the intention-behavior gap in SCB in another cultural context.

### Practical implications

The results of this study have important practical implications for the promotion of SCB. *First,* the results suggest that interventions aimed at promoting SFC should focus on fostering values that prioritize the environment, personal and collective well-being, and personal growth. For example, campaigns highlighting the environmental and social benefits of SFC may appeal to individuals who hold these values. Furthermore, the findings imply that interventions aimed at promoting SFC should focus not only on increasing awareness of the consequences of unsustainable fashion consumption but also on promoting a sense of responsibility for one’s actions. Such interventions could include educating individuals on the environmental impact of their fashion choices and encouraging them to take responsibility for their actions towards the environment to adopt a new ecological paradigm. This can be achieved through educational campaigns and by creating a sustainable fashion culture that highlights the importance of SCB. These interventions can be implemented through various channels, such as social media campaigns, educational programs, and fashion industry initiatives that promote SFC.

*Second,* this study emphasizes the importance of creating social norms that support SFC., which can be achieved through initiatives such as sustainable fashion events, sustainable fashion collaborations, and sustainable fashion blogs. By creating a sustainable fashion community that promotes SCB, individuals are more likely to adopt this behavior. *Third,* this study highlights the importance of increasing TR, which is a significant predictor of SCB and has important implications for policymakers and marketers seeking to promote SFC. This can be achieved through initiatives that increase awareness about the importance of recycling and highlight the benefits of recycling, such as reduced waste and increased resource efficiency. Additionally, initiatives that increase access to recycling facilities and make recycling more convenient can increase TR and promote SCB.

*Finally,* this study provides important insights into the intention-behavior gap in SCB. These findings suggest that SCI and TR are significant predictors of SCB. Therefore, interventions aimed at promoting SFC should focus on increasing individuals’ SCI and TR to close the intention-behavior gap and encourage SCB.

## Conclusion

This study highlights the significant roles played by values, attitudes, and norms in shaping SCB among individuals. These findings suggest that individual values, including BV, AV, and EV, significantly influence EP, which positively affects AC. This awareness leads to a greater sense of responsibility toward the environment, which, in turn, positively influences SCB. This study also suggests that PN plays a vital role in shaping SCB, and creating an environment where SFC is considered normal, to encourage more individuals to adopt SCB. Moreover, this study emphasizes the importance of increasing TR to promote SCB. The findings have significant implications for policymakers and businesses aiming to promote SFC and create a more sustainable fashion industry.

This study has limitations worth noting. Firstly, it exclusively examined individual-level factors influencing SCB and did not consider broader societal influences like policies and regulations that might impact SCB. Future research should explore the interplay between individual and societal factors to achieve a more comprehensive understanding of SCB. Another limitation is the study's neglect of other factors like price, availability, and convenience that can affect SCB. While values, beliefs, and norms are important predictors of behavior, practical factors also play a role in individuals' engagement in SFC. Subsequent research could assess the relative influence of these practical factors in addition to values, attitudes, and norms. Additionally, the study concentrated on SCB without examining the influence of sustainable fashion production practices. Sustainable production practices, including the use of sustainable materials, waste reduction, and fair labor practices, are crucial for overall sustainable fashion. Future research should explore the factors affecting these production practices and their relationship with consumption.

Furthermore, the study relied on self-reported SCB measures, potentially subject to social desirability bias, wherein participants might have overreported SCB to appear more socially responsible. Future research could employ objective measures, like tracking actual purchasing and disposal behaviors. The study used a cross-sectional design, limiting its ability to establish causality between the variables studied. Future research could utilize longitudinal or experimental designs to investigate causal relationships between variables. Finally, the present study adopted the convenience sampling method, which might trigger common method biases, even though precautions were taken during the study. Advanced research could consider employing other sampling techniques, such as probability sampling, to mitigate any potential biases and ensure more accurate results.

### Supplementary Information

Supplementary Information 1.

Supplementary Information 2.

## Data Availability

The original contributions presented in the study are included in the article/Research Data, further inquiries can be directed to the corresponding author/s.
