# Supplementary material for: Predicting sustainable fashion consumption intentions and practices
Source: Sci Rep. 2024 Jan 19;14:1706. doi: 10.1038/s41598-024-52215-z (PMC10799023; doi:10.1038/s41598-024-52215-z)
Supplement: Supplementary file 1 — Supplementary Information 1. [file 41598_2024_52215_MOESM1_ESM.docx]

**S1.** Survey Instrument

| Code | Items | | |
| --- | --- | --- | --- |
| BV1 | Preventing pollution | | |
| BV2 | Respecting the earth | | |
| BV3 | Unity with nature | | |
| BV4 | Protecting the environment | | |
| AV1 | Equality | | |
| AV2 | A world at peace | | |
| AV3 | Social justice | | |
| AV4 | Helpful | | |
| EV1 | Social power | | |
| EV2 | Wealth | | |
| EV3 | Authority | | |
| EV4 | Influential | | |
| EV5 | Ambitious | | |
| EP1 | When humans interfere with nature, the consequences can be disastrous | | |
| EP2 | Plants and animals have as much right to live as humans | | |
| EP3 | Conventional food production is seriously abusing the environment | | |
| EP4 | The balance of nature is very delicate and easily upset | | |
| EP5 | Human is responsible for the long-life of nature sustainability | | |
| AC1 | Global warming is a problem for society | | |
| AC2 | Recycling help reduce global warming | | |
| AC3 | Environmental quality will improve if we practice sustainable fashion consumption | | |
| AC4 | Protection of the environment benefits us all | | |
| AC5 | Sustainable fashion consumption is beneficial for our community | | |
| AC6 | Sustainable fashion consumption improves our quality of life | | |
| AR1 | We are jointly responsible for global warming | | |
| AR2 | We are jointly responsible for the environmental problems caused by the fossil fuel industry | | |
| AR3 | We are jointly responsible for the environmental problems caused by conventional fashion consumption | | |
| AR4 | We are jointly responsible for the environmental deterioration caused by conventional fashion consumption | | |
| AR5 | We are jointly responsible for the ecological deterioration caused by conventional fashion consumption | | |
| PN1 | I feel morally obliged to reduce fashion waste | | |
| PN2 | People like me should do everything they can to reduce fashion waste | | |
| PN3 | I feel obliged to bear the environment and nature in mind in my consumption behaviours | | |
| PN4 | I feel morally obliged to practice sustainable fashion consumption, regardless of what others do | | |
| PN5 | I feel personally obliged to practice sustainable fashion consumption | | |
| SN1 | Family members whose opinion I value would approve of my sustainable fashion consumption practices | | |
| SN2 | Family members whose opinion I value would approve of my engagement in recycling fashion products | | |
| SN3 | Most people who are important to me think I should do whatever I can to reduce fashion waste | | |
| SN4 | Most people who are important to me would want me to take action to stop conventional fashion consumption | | |
| SN5 | People whose opinions I value would prefer that I do whatever I can to prevent the conventional fashion consumption | | |
| TR1 | | Recycling fashion products are generally reliable | |
| TR2 | | Recycled fashion product’s environmental performance is generally dependable | |
| TR3 | | Recycled fashion product’s environmental argument is generally trustworthy | |
| TR4 | | Recycled fashion product’s environmental concern meets your expectations | |
| TR5 | | Recycling method’s keep promises for environmental protection | |
| SCI1 | All things considered, I want to sell and/or swap /donate second-hand clothing online often in the future. | | |
| SCI2 | I can see myself engaging in selling and/or swapping/donating second-hand clothing in the future. | | |
| SCI3 | I can see myself selling and/or swapping/donating second hand clothing if possible. | | |
| SCI4 | I will likely frequently sell and/or swap /donate second-hand clothing in the future. | | |
| SCI5 | It is very likely that I will sell and/or swap /donate second-hand clothing in the future. | | |
| SCB1 | | I help to reduce second-hand clothing waste by selling, swapping, and/or donating used clothing. |  |
| SCB2 | | I sell, swap, and/or donate second-hand clothing to reduce the waste of resources. |  |
| SCB3 | | I sell, swap, or donate second-hand clothing to dispose of it. |  |
| SCB4 | | I sell, swap, and/or donate second-hand clothing to cut costs. |  |
| SCB5 | | I sell, swap, and/or donate second-hand clothing to reduce environmental degradation. |  |

**Note:** BV - Biospheric Values; AV - Altruistic Values; EV - Egoistic Values; EP - New Ecological Paradigm; AC – Awareness of Consequences; AR - Ascription of Responsibility; PN - Personal Norms; SN – Social Norms, GR - Trust in Recycling; SCI - Sustainable Fashion Consumption Intention; SCB - Sustainable Fashion Consumption Behaviour
